# Acute Effects of Post‑Activation Performance Enhancement of 5RM Weighted Pull‑Ups and One Arm Pull‑Ups on Specific Upper Body Climbing Performance

**DOI:** 10.2478/hukin-2022-0097

**Published:** 2022-11-08

**Authors:** Krzysztof Sas-Nowosielski, Klaudia Kandzia

**Affiliations:** 1 Institute of Sport Sciences, The Jerzy Kukuczka Academy of Physical Education in Katowice, Katowice, Poland

**Keywords:** sport climbing, upper body power, post-activation performance enhancement

## Abstract

This study aimed to compare the acute effects of performing two kinds of pull-ups: traditional, pronated grip pull-ups performed with two arms and additional weight with loading intensity of 5RM and one-arm pull-ups, on specific upper body climbing power. Twenty-four advanced climbers participated in the study. The International Rock Climbing Research Association (IRCRA) Power Slap Test was chosen to assess specific upper body climbing power. All athletes performed the test under three conditions: control (without a conditioning activity) and both kinds of pull-ups as conditioning activities. Results revealed significant improvements in the Power Slap's distance, power, velocity, and force in 5RM weighted pull-ups, but not in one-arm pull-ups. In the latter case, participants reached higher power values after the conditioning stimulus, but the effect size was small. Also, the differences with the remaining variables (power, speed, and force) were non-significant. The results suggest that weighted pull-ups with a 5RM intensity and not one arm pull-ups seem to be an effective PAPE stimulus. Therefore, the former can be used as a conditioning activity before an explosive climbing exercise such as the Power Slap on a campus board.

## Introduction

Rock and sport climbing have increased in popularity, with the latter entering the Olympic Games schedule in Tokyo 2020 and Paris 2024. As a sport discipline, climbing involves three kinds of events: Lead, Speed, and Bouldering. Each of them imposes slightly different physiological requirements on participants. Still, repetitive forceful muscle contractions are required to move the climber’s body on the climbing wall in all events.

This tendency is most visible in bouldering, which involves climbing short, barely a few meters long routes called ‘problems’. They vary in nature, but in almost all competitions, at least one of the problems in a given round requires the athlete to use the so-called ‘dynamic techniques’, like ‘dynos’ and ‘monos’. In climbing terminology, they refer to techniques for moving between holds that require the athlete to jump from hold to hold. Such techniques also have a significant share in speed climbing. The distances between holds and the speed at which competitors are currently moving (the current world record is 5.208 s on the 15 m high wall) changed climbing the wall into a series of jumps between holds.

Changes taking place within sport climbing pose new challenges for athletes. As never before, the success of their competitive efforts depends on their ability to unleash high power output and explosive strength, i.e., to increase force or torque as quickly as possible during a rapid voluntary contraction (Maffiuletti et al., 2016).

The shift in emphasis of the climbers' physical preparation towards developing larger forces in a shorter time has prompted athletes and coaches to search for new means and methods of training, previously rarely used in this community. Among the various forms of power development that may prove helpful in sport climbers’ conditioning there is complex training, which involves the simultaneous application of different training measures in the same training unit or microcycle (Verkhoshansky and Siff, 1999; Šinkovec and Rugelj 2020). Within a single training session, this differentiation mainly refers to selecting biomechanically similar exercises, used in the following sequence: resistance exercise followed by a plyometric, a ballistic, or a speed exercise. The most popular pairs of activities include squats and jumps, squats and sprints, bench press and clap push-ups, shoulder presses and overhead medicine ball throws (Harrison et al., 2019; Seitz and Haff, 2016). Such exercise sequences result in a temporal increase in power and force production, thus allowing more significant training stimuli and/or enhancing acute performance (Docherty and Hodgson, 2007).

The physiological rationale for complex training is a phenomenon known as post-activation potentiation (PAP) or post-activation performance enhancement (PAPE) (Blazevich and Babault, 2019; Boullosa et al., 2020). Tillin and Bishop (2009) defined it as ‘acute enhancement of muscular performance characteristics as a result of their contractile history. The exact nature of PAPE is not well understood. Several mechanisms were proposed to explain its effect on performance: increasing neural excitability (better motor-unit recruitment and synchronization, decreased presynaptic inhibition), an increased amount of Ca^2+^ in the sarcoplasmic reticulum and greater sensitivity of the myofilaments to Ca^2+^, reduction in the sensitivity of Golgi tendon organs and Renshaw cells thus weakening their inhibitory actions, changes in muscle architecture, and especially a decrease in the pennation angle of muscle fibres with a resultant increase of forces that are transferred onto the bones (Docherty and Hodgson, 2007; Sas-Nowosielski and Kaczka 2022; Scott and Docherty, 2004; Tillin and Bishop, 2009).

Regardless of the true nature of PAPE, it seems to induce acute and long term effects on performance in various lower- and upper body activities such as jumps and sprints as well as selected upper body exercises including the bench press and bench press throw (Duthie et al., 2002; Docherty and Hodgson, 2007; Krzysztofik et al., 2020a, 2021; Liossis et al., 2013; Loturco et al., 2014). To our knowledge, only Gołaś et al. (2016) and Sas-Nowosielski and Kandzia (2020) investigated PAPE in the upper-body exercises that also involved ‘pulling’ movements. In the Gołaś et al.’s (2016) study, these movements were lat pull-downs and dumbbell rows. Since the kinematic characteristics of these exercises are different from the essential motor activities occurring in climbing, Sas-Nowosielski and Kandzia (2020) investigated the effect of weighted pull-ups on the effectiveness of the Power slap test recommended by the International Rock Climbing Research Association (IRCRA) for power assessment in climbing and being also one of the most popular explosive strength and power exercises on the campus board (Michailov, 2014). The results showed that post-baseline slap distances were significantly greater in the experimental group, while no changes were observed in the controls. The limitation of this study was the inference of PAPE effects on the reach distance achieved by the subjects in the test without recording other movement variables.

Furthermore, weighted pull-ups may not be practical as a strengthening stimulus before the competition, as the warm-up zone contains a climbing-specific device (climbing walls, fingerboards), but not necessarily free weights. Under these conditions, an alternative strength exercise that athletes can perform under almost any conditions and at the same time meet the requirements of motor similarity to the target activity is the one-arm pull-up. However, this possibility has not been considered in research so far. Taking into account the above, the purpose of this study was to investigate the acute effects of two conditioning activities: weighted pull-ups and one-arm pull-ups on performance of explosive activity (Power slap). We hypothesized that both conditioning activities would acutely enhance explosive pull-up (Power slap) performance.

## Methods

### Participants

A total of 24 climbers, (mean ± SD): age: 30.9 ± 7.2 years; body height: 173.6 ± 6.4 cm; body mass: 65.0 ± 9.6, were recruited for this study. All participants were advanced climbers. Their climbing performance level was determined based on self-reported best red-point (RP) and on-sight (OS) climbs. After being transformed into the IRCRA reporting scale (Draper et al., 2011) it was 22.0 ± 4.0 and 17.9 ± 3.5, respectively. According to the classification system adopted by the IRCRA for describing individual climbers and group abilities, the study participants were advanced climbers. As they all had experience in campus board and weighted pull-up exercises, familiarization sessions were not included in the present study. Participants were informed of the aims and procedures of the study before taking part in the investigation. One of the researchers demonstrated the Power Slap test to familiarise participants with the test procedure, particularly with the beeps produced by the Gyko sensor. The study was conducted 24 h after the athletes' last practice, allowing the neuromuscular system and skin on the fingertips to recover. Before testing, participants were instructed to perform a warm-up consisting of climbing circuits on a bouldering wall followed by a set of dynamic pull-ups on campus rugs. However, the exact number of sets and repetitions was not imposed, as it was decided to rely on the climbers' personal experience and their different preferences for warming up before exercising on the campus board. This allowed athletes to create optimal conditions to prepare their muscles and fingers for practice on the campus board. We were also guided by the highly subjective nature perceptions of climbing effort. For this reason, imposing the same number and type of climbing moves may have differed from the abilities of individual climbers and may have resulted in an inadequate warm-up for some and excessive fatigue for others.

### Design and Procedures

**Figure 1:**
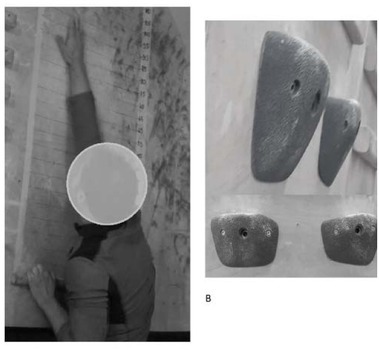
Power slap (A), and holds used in the study (B).

The Power Slap (IRCRA, 2015; Draper et al., 2011), chosen as a power test, was performed on a board on which a scale with distances in centimetres was drawn. At the bottom of the board, a pair of climbing holds (Crimps M by BluePill) was screwed. Both grips had a folding surface allowing the fingers to be curled over the lip of the hold and a coarse texture to limit the risk of the fingers slipping out of the grip during the test, which could affect performance (Figure 1). Independently, participants were allowed to dry their fingers with chalk (magnesium carbonate) before the test. As in the Laffaye et al.’s (2014) study, the holds were spaced 55 cm apart, as an optimal spacing, within the range of upper-limb optimal strength. According to the IRCRA recommendations, the manual climber’s task was to hold on to the rung with straight arms, initiate an explosive pull-up, and slap as high as possible with the right arm. The performance was measured by establishing a point on a scale touched by the climber’s hand. To ensure greater accuracy of the distance readings, each climber was video-recorded.

### Instruments

The Gyko inertial sensor (dimensions: 53 × 51 × 23 mm, mass: 46 g) (Microgate, Bolzano, Italy) was used to register velocity (m/s), force (N), power (W) of pull-ups, and calculation of 1RM. The device contains a three-dimensional accelerometer (range: ±2 G), a gyroscope (250°/s-25000°/s), and a magnetometer (range: ±4800 μT). It allows recordings at a sampling frequency of 1 kHz. Participants had the Gyko sensor attached at the level of the centre of the body mass on the back using an elastic belt provided with the sensor. During measurements, the signals were transferred via Bluetooth 4.0 to a Lenovo PC with installed REPOWER software, following the criteria described by the manufacturer.

### Procedure

A crossover study design was used. All participants performed a control (no PAPE stimulus) and two experimental trials (after 5RM weighted pull-ups and one arm-assisted pull-ups). The individual trials were planned at weekly intervals. Due to the outbreak of the third wave of coronavirus and the associated restrictions, the third series of trials was conducted one month after the second series. Due to the ongoing restrictions, some participants could not participate in all three trials; thus the size of particular groups changed (n = 24 control, n = 18 PAPE 5RM, n = 14 one arm). The load to perform the 5RM was defined based on the current results of fitness tests conducted by coaches and/or training diaries of athletes. In situations when climbers invited to participate in the study did not perform targeted strength exercises in the form of weighted pull-ups or one-arm pull-ups, they were asked to determine these values approximately one week before the planned tests. Both in the first and the second exercise athletes gripped the bar with an extended grip, with fingers interlocked, thus making the grip of the bar similar to the grip used during the hang slap test.

Under control conditions, participants performed two Power Slaps separated by 4 min rest intervals, 8-10 min after the warm-up. After the first trial, participants performed one of the two PAPE activation exercises and, after a 4-min rest interval, the second Power Slap in both experimental trials. The 4-min rest interval was chosen for two reasons. Firstly, according to Lowery et al. (2012) and Wilson et al. (2013), 4-8 min rest intervals are close to optimal. Secondly, 4 min is a typical rotation time in bouldering competitions, and while establishing the study protocol, we had to bear in mind the practical aspects of our research. The 5RM load values were determined for each participant based on their training programs and ranged between 9 and 47 kg, averaging 27.8 ± 10.1 kg and representing 15 to 70% of body mass (M = 41.9 ± 15.3%). Participants performed pull-ups on a horizontal bar being instructed to perform the pull-ups starting with their arms fully extended to a position in which the chin reached the bar level. Pull-ups were to be executed in a row without stopping.

The one-arm pull was performed from a straight arm position to a full arm flexion until the chin was at the height of the bar or touched the hand holding the bar. There is a wide variation among climbing athletes regarding their skills and abilities to perform this exercise, i.e., from zero to dozen or more repetitions. Therefore, this exercise was performed in two variations. Participants (n = 2) who could perform 4-5 repetitions of this exercise without assistance performed it in this form. Weaker ones performed this in an assisted form, i.e., using an elastic silicone band, allowing weight relief. The band was caught at such a height that the arm with the forearm formed an angle of 35-40%. The band's elasticity was adjusted so that the participant could perform between 3 and 5RM, one set for each arm.

The Ethics Committee approved the study protocol of Biomedical Research at the Academy of Physical Education in Katowice – resolution no 1/2019.

### Statistical Analysis

Descriptive statistics (means, standard deviations, and 95% confidence intervals for mean values) were used to describe the data. Assumptions of normality and homogeneity of variance were tested with the Shapiro–Wilk and Levene’s tests, respectively. Repeated measures ANOVA with a Tukey post hoc test was used to assess the effects of the pre-post PAPE. As a measure of effect size between both conditions, Cohen’s *d* was calculated, and the obtained values were interpreted as recommended by Cohen as: ‘trivial’ *d* < 20, ‘small’ *d* = 20–49, moderate’ *d* = 50– 79, and ‘large’ *d* > 80 (Lakens, 2013). Statistical significance was accepted at *p* ≤ 0.05. All analyses were conducted using Statistica 13.3 (Statsoft, Cracow, Poland) software, while the effect sizes were determined using the Stats.xls calculator (missouristate.edu/rstats accessed on October 21, 2019).

## Results

Table 1 presents the main results of the Power Slap for each procedure.

**Table 1:** Power slap scores across all tested protocols.

	Mean ± SD	95% CI	*p* value	Effect size	
**No PAPE**					
Slap distance 1 (cm)	86.3 ± 8.8	82.6–90.1			
Slap distance 2 (cm)	87.3 ± 9,0	83.5–91.1	0.063	0.11	Trivial
Power 1 (W)	503.6 ± 114.3	455.4–551.9			
Power 2 (W)	510.4 ± 113.1	462.7–558.2	0.607	0.06	Trivial
Velocity 1 (m∙s^–1^)	0.8 ± 0.2	0.7–0.9			
Velocity 2 (m∙s^–1^)	0.8 ± 0.2	0.7–0.9	0.211	0.02	Trivial
Force 1 (N)	859.5 ± 163.3	790.5–928.4	0.898	0.01	Trivial
Force 2 (N)	861.4 ± 142.9	801.1–921.8			
**5RM weighted pull–ups**					
Slap distance pre (cm)	84.0 ± 11.2	78.4–89.5	<0.001	0.68	Moderate
Slap distance post (cm)	91.4 ± 10.6	86.2–96.7			
Power pre (W)	501.3 ± 92.7	455.2–547.4			
Power post (W)	585.3 ± 158.2	506.6–664.0	<0.001	0.52	Moderate
Velocity pre (m∙s^–1^)	0.8 ± 0.1	0.7–0.8	0.002	0.80	Moderate
Velocity post (m∙s^–1^)	0.9 ± 0.2	0.8–1.0			
Force pre (N)	823.9 ± 114.4	767.1–880.8	<0.001	0.59	Moderate
Force post (N)	896.7 ± 135.4	829.4–964.0			
**One arm pull–ups**					
Slap distance pre (cm)	86.9 ± 6.8	83.0–90.8			
Slap distance post (cm)	89.8 ± 7.3	85.6–94.0	0.001	0.41	Small
Power pre (W)	577.0 ± 131.4	501.2–652.8			
Power post (W)	587.7 ± 139.6	507.1–668.3	0.341	0.08	Trivial
Velocity pre (m∙s^–1^)	0.9 ± 0.2	0.8–1.0	0.888	0.02	Trivial
Velocity post (m∙s^–1^)	0.9 ± 0.2	0.8–1.0			
Force pre (N)	857.9 ± 227.7	726.4–989.3			
Force post (N)	886.0 ± 214.1	762.4–1009.6	0.158	0.13	Trivial

No significant differences were found in distance (F_(1, 23)_ = 3.81, *p* = 0.063), power (F_(1, 23)_ = 0.27, *p* = 0.607), velocity (F_(1, 23)_ = 1.66, *p* = 0.211) and force (F_(1, 23)_ = 0.02, *p* = 0.898).

The distance of the Power Slap between the first and second trials differed significantly in the 5RM pull-ups PAPE condition, F_(1, 17)_ = 67.54, *p* < 0.001, averaging 7.4 cm between trials. A significant improvement between pre-PAPE and post PAPE was also observed in power (F_(1, 17)_ = 23.85, *p* < 0.001), velocity (F_(1, 17)_ = 12.71, *p* = 0.002) and force (F_(1, 17)_ = 17.34, *p* < 0.001). An insight into individual climbers' performance revealed that all participants improved in the 5RM condition by achieving a greater distance in the slap test and the rest of the measured variables. The magnitude of the differences was not affected by the value of the load constituting the PAPE stimulus, either absolute (the amount of weight with which the participant performed the 5 pull-ups) or relative (the ratio of weight magnitude to body mass). None of the regression models performed with the load magnitude as predictors and distance, power, and strength as dependent variables was statistically significant: corr. R^2^ = 0. 08, F_(2,14)_ = 1.74, *p* = 0.211; corr. R^2^ = 0.13, F_(2,14)_ = 0.10, *p* = 0.906; corr. R^2^ = 0.08, F_(2,14)_ = 1.74, *p* = 0.211 and corr. R^2^ = 0.13, F_(2,14)_ = 1.07, *p* = 0.371, respectively.

In one-arm pull-ups a significant difference was found in the distance of the Power slap (F_(1, 13)_ = 16.20, *p* = 0.001), but not in the remaining variables, i.e., power (F_(1, 13)_ = 0.978, *p* = 0.341), velocity (F_(1, 13)_ = 0.020, *p* = 0.888) and force (F_(1, 13)_ = 2.24, *p* = 0.158). The magnitude of the difference in the distance was small (*d* = 0.41), averaging 2.9 cm. Figure 1 illustrates individual responses in all three conditions, comparing baseline distances, power, and force to post distances, power, and force.

**Figure 2:**
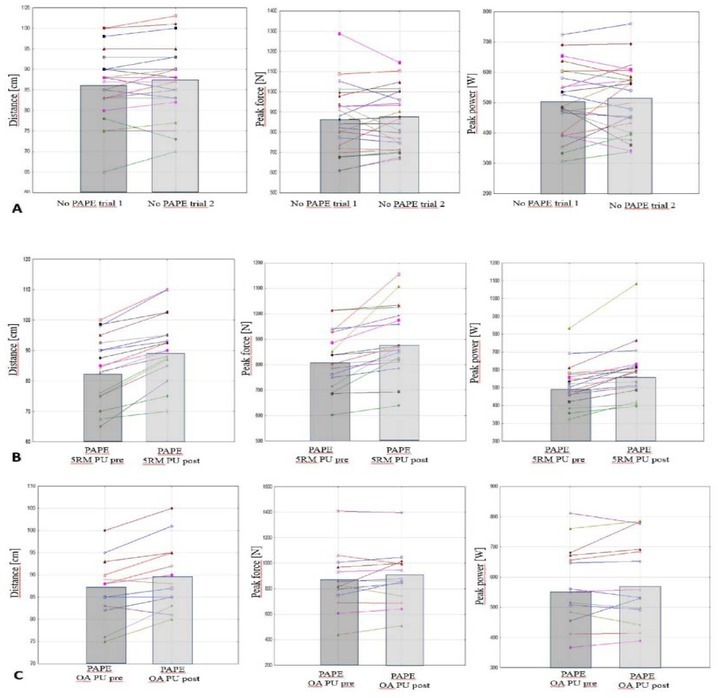
Individual response (lines) and group mean (bars) of Power slap test measures: distance [cm], peak force [N] and peak power output [W]. A – control, no-PAPE, B – PAPE 5RM weighted pull-ups (5RM PU), C – PAPE one-arm pull-ups (OA PU).

## Discussion

To the best of the authors’ knowledge, this study is the first to investigate the acute effects of two kinds of pull-ups performed on a campus board on climbing-specific power (Power slap). The study's theoretical basis was the PAPE phenomenon in which ‘muscular performance characteristics are acutely enhanced due to their contractile history’ (Tillin and Bishop, 2009). The ‘contractile history’ usually means an exercise of maximal or near-maximal muscular contraction, called the conditioning activity, followed by movement or activity that requires a rapid expression of force or power (de Freitas et al., 2021). Previous studies have reported a possible ergogenic effect of PAPE on acute performance and chronic conditioning (Docherty and Hodgson, 2007; Helena et al., 2019; Krzysztofik et al., 2020b; Tillin and Bishop, 2009; Wilson et al., 2013). However, most of them were conducted on pairs of lower body activities such as squats and vertical jumps, squats, resisted sprints and sprints etc. (Beato et al., 2019; Dobbs et al., 2019; Lagrange et al., 2020; Matusiński et al., 2021; Tillin and Bishop, 2009). Fewer studies have focused on the effects of PAPE on upper body explosive performance (Ebben, 2002). Those who have addressed this issue tended to include activities involving 'pushing', such as bench presses and ballistic push-ups (Farup and Sørensen, 2010; Liossis et al., 2013; Seitz and Haff, 2016). For that reason, their findings cannot be directly translated into climbing in which ‘pulling’ movement patterns predominate and many of which require high rates of force development. Viewed from this perspective, PAPE offers the potential to support many efforts in climbing. Previous research has confirmed the positive effect of 5 RM pull-ups on the distance achieved in the Power slap (Sas-Nowosielski and Kandzia, 2019). The current study confirmed previous results, enriching them with more direct measurements of power and force. The intensity of the pull-ups was 5RM, which corresponded to 85-87% of 1RM, i.e., the intensity within the range considered as the most effective for eliciting PAPE (Carter and Greenwood, 2014). In light of the results discussed above, weighted pull-ups performed 4 min before the 'power slap' appear to be a helpful strategy in eliciting acute enhancement of climbing-specific upper body power. However, although useful in training conditions, this exercise may be of little use under competition conditions in the absence of free weights in the warm-up zone. Therefore, our next objective was to evaluate whether the one-arm pull-ups could be an alternative PAPE stimulus.

Contrary to our expectations, the current findings rejected the hypothesis on potentiating effect of one-arm pull-ups. Although participants managed to reach an average of about 3 cm further and obtained slightly higher values of power and force, only changes in distance were statistically significant. Concerning the magnitude of differences based on effect size calculations, change in the slap distance were negligible, while differences in power, force and velocity were trivial. One of the possible explanations for the observed significant improvement in the distance with non-significant differences in the other movement variables (force, power, velocity) may relate to the stretching of the passive elements of the musculoskeletal system taking place during the execution of the overhang and then the pull-ups on one arm. The distance improvement could also result from a slight change in the movement technique, at least in some athletes (even a slight rotation of the shoulders along the long axis of the body increases the arm's reach). It can be concluded that a conditioning stimulus of one arm pull-ups was not very effective in enhancing Power slap performance.

A significant inter-individual variation in response to PAPE was observed in our study. For the 5RM weighted pull-ups, all participants responded with improved Power slap performance, while for the one-arm pull-ups, the individual reactions ranged from worse to enhanced performance. An explanation for this variation is not possible in light of the data collected in the present study. Previous research has revealed that a variable that may influence the magnitude of responses is the level of strength. Stronger individuals may exhibit a more robust PAPE response than their weaker counterparts (Seitz et al., 2014; Seitz and Haff, 2016; Tillin and Bishop, 2009). The former usually have a greater percentage of type II muscle fibers and greater phosphorylation of the myosin light chain proposed as one of the mechanisms underpinning PAPE. In our study, at least for the weighted pull-ups, we did not find that differences in the strength level affected the magnitude of the PAPE response. This variable is more difficult to control concerning the one-arm pull-ups, especially in individuals who perform this exercise in an assisted form. In future studies, it would be worthwhile to compare the PAPE response in subjects performing one-arm pull-ups in unassisted and assisted forms and also to determine the magnitude of relief more precisely, perhaps based on the velocity of the movement known to be related to RM magnitude (González-Badillo and Sánchez-Medina, 2010; Loturco et al., 2021; Pérez-Castilla and García-Ramos, 2020). The one-arm pull-up is slightly different in movement structure compared to the power slap, and despite the involvement of the same muscle groups, muscle activation patterns may slightly vary (Dickie et al., 2017; Kozin et al., 2020; Leslie and Comfort, 2013). Considering the criterion of biomechanical similarity between complementary exercises (resistance exercise - explosive exercise), weighted pull-ups performed in the traditional two arms manner may be an exercise that fulfils this condition more fully than one-arm pull-ups. However, doubts of this nature are an open field of research.

It is plausible that several limitations might have influenced the results of the study. First of all, the one-arm pull-up is an exercise for which intensity is difficult to assess, whether it is evaluated as a certain percentage of a one-repetition maximum (%RM) or a resistance that allows performing a specified number of repetitions (RM). However, as far as the authors are aware, no study has assessed one-arm pull-ups so far. Secondly, we decided on only one set of conditioning exercises, one of many possible options. The number of sets and repetitions determines the size of the stimulus, which can induce PAPE, but also affects the level of fatigue that coexists with it. The magnitude of the stimulus should therefore be chosen so that the potentiating effects outweigh the fatigue. However, there is no consensus on what this might mean in practice, and previous studies have used both single and multiple sets of exercises (Seitz and Haff, 2016). It is worthwhile to contrast the effects of different numbers of sets on the power slap in future research. Thirdly, it is also possible that the optimum time interval may be different for both types of exercise; thus, in future studies, various rest intervals should be assessed. Fourthly, despite recording each participant on video, the reading accuracy may not have been ideal, at least in some cases. Another limitation may be related to the device we used to measure power, force, and movement velocity. Gyko belongs to the category of linear transducers, which primarily measure the vertical component of the velocity vector, failing to record movements and their horizontal components, which in movements with an existing horizontal component can cause errors in power estimation (McBride, 2017). Pull-ups constitute an example of exercise in which the vertical displacement of the centre of gravity is dominant; however, there may be a slight horizontal displacement of the body's centre of gravity during the initial movement phase. Climbers were asked to assume a stable starting position and begin the pull-up without additional trunk movement to minimise this type of interference. Despite this, some errors in the estimation of the measured variables cannot be excluded.

## Conclusion

Despite the presented limitations, we are convinced that our study is one of the first on the ad hoc effects of PAPE in climbing, and has some implications for climbing training and the warm-up before competitions. From our data it can be concluded that the use of weighted pull-ups of 5RM intensity provides a better stimulus for inducing PAP responses than one-arm pull-ups.
